# Influence of Nanoparticle Processing on the Thermoelectric Properties of (Bi_x_Sb_1−X_)_2_Te_3_ Ternary Alloys

**DOI:** 10.1002/open.202000257

**Published:** 2021-01-25

**Authors:** Sarah Salloum, Georg Bendt, Markus Heidelmann, Kateryna Loza, Samaneh Bayesteh, M. Sepideh Izadi, Patrick Kawulok, Ran He, Heike Schlörb, Nicolas Perez, Heiko Reith, Kornelius Nielsch, Gabi Schierning, Stephan Schulz

**Affiliations:** ^1^ Institute for Inorganic Chemistry and Center for Nanointegration Duisburg-Essen (Cenide) University of Duisburg-Essen Universitätsstraße 5–7 45117 Essen Germany; ^2^ Institute for Metallic Materials Leibniz Institute for Solid State and Materials Research Dresden Helmholtzstrasse 20 01069 Dresden Germany; ^3^ Institute of Applied Physics Dresden University of Technology 01069 Dresden Germany; ^4^ Institute of Materials Science Dresden University of Technology 01069 Dresden Germany; ^5^ Interdisciplinary Center for Analytics on the Nanoscale (ICAN) NETZ University of Duisburg-Essen Carl-Benz-Str. 199 47047 Duisburg Germany; ^6^ Faculty of Physics University of Bielefeld 33501 Bielefeld

**Keywords:** bismuth antimony telluride, nanoparticles, electrical conductivity, Seebeck coefficient, thermal conductivity

## Abstract

The synthesis of phase‐pure ternary solutions of tetradymite‐type materials (Bi_x_Sb_1−x_)_2_Te_3_ (x=0.25; 0.50; 0.75) in an ionic liquid approach has been carried out. The nanoparticles are characterized by means of energy‐dispersive X‐ray spectroscopy (EDX), powder X‐ray diffraction (PXRD), scanning electron microscopy (SEM), and transmission electron microscopy. In addition, the role of different processing approaches on the thermoelectric properties ‐ Seebeck coefficient as well as electrical and thermal conductivity ‐ is demonstrated.

## Graphical abstract

**Tetradymite‐type ternary** (Bi_x_Sb_1‐x_)_2_Te_3_ (x=0.25, 0.5, 0.75) nanoparticles were synthesized in an ionic‐liquid approach. The effect of thermal annealing on the chemical composition and morphology of the nanoparticles as well as their transport properties prior and after annealing is reported.

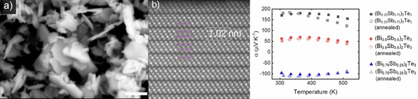

## 1 Introduction

Today, the alarming risks of global warming and energy shortage continue to rise with the depletion of unsustainable energy sources such as coal, petroleum, and natural gas. Despite an increasing demand for energy, considerable amounts of untapped energy in the form of waste heat derive from almost every electrical and mechanical process, including machine operation, oil refining, steel making and food production. In recent years, waste‐heat harvesting and recovery using thermoelectric (TE) materials have attracted significant attention as a promising technology to generate clean energy and reduce carbon emission.^[1]^ By definition, TE materials are capable of converting heat to electricity via the Seebeck effect, and electrical energy into cooling via the Peltier effect. TE materials can therefore be used in a wide range of technical applications which include power generators, cooling devices, IR detectors, and gas sensors.[^2^, ^3^, ^4^, ^5^]

The energy conversion efficiency of a thermoelectric material is typically represented by the dimensionless figure‐of‐merit *zT=(α^2^σT/κ)*, where α is the Seebeck coefficient, σ the specific electrical conductivity, T the absolute temperature in Kelvin, and κ the thermal conductivity as the sum of the electronic κ_el_ and lattice κ_L_ contributions.[^1^, ^6^, ^7^, ^8^, ^9^] In order to achieve a high thermoelectric figure‐of‐merit, the material of interest must exhibit a sufficiently large Seebeck coefficient, a high electrical conductivity, and a low thermal conductivity. Notably, antimony (Sb_2_Te_3_) and bismuth telluride (Bi_2_Te_3_) as well as their solid ternary solutions (Bi_x_Sb_1−x_)_2_Te_3_ have already found their way in many commercialized thermoelectric devices due to their remarkable performance near room temperature.[^10^, ^11^, ^12^, ^13^, ^14^, ^15^, ^16^] These compounds belong to a class of so‐called tetradymite‐type semiconductors which exhibit heavy doping and narrow band gap characteristics, as well as a unique quintuple‐layered structure.[^12^, ^13^, ^16^, ^17^, ^18^, ^19^] Despite their high potential as thermoelectric candidates, group V chalcogenides have a strong tendency to generate anti‐site defects due to the spontaneous occupation of the Te lattice sites by Sb and Bi atoms. The occurrence of non‐stoichiometric compositions reduces the Seebeck coefficient, and hence has limited existing *zT* values to less than 1.[^20^, ^21^, ^22^] Though many efforts were made to overcome such intrinsic limitations and optimize the *zT* value,[^23^, ^24^, ^25^] the interdependence of the thermoelectric transport properties renders it rather difficult to enhance one physical parameter without diminishing the others.[^26^, ^27^, ^28^] Interestingly, nanostructuring (i. e. nanograins and metallic nano‐inclusions) as well as point defect engineering are long‐proven strategies that have helped achieve significant optimization of thermoelectric materials by decoupling the transport parameters. Such phenomena selectively reduce the lattice thermal conductivity in nanostructured materials such as nanoparticles and thin films due to the additional scattering of heat carrying phonons at grain boundaries and interfaces.[^9^, ^11^, ^25^, ^27^, ^29^, ^30^, ^31^, ^32^, ^33^] As a result, the synthesis of chalcogenide‐based nanostructures of various shape and size has been widely studied using a variety of methods such as electrochemical deposition, solvothermal or hydrothermal approaches[^18^, ^34^, ^35^, ^36^] as well as microwave‐assisted approaches,^[37]^ mechanical alloying,^[38]^ and gas phase processes (i. e. atomic layer deposition ALD,[^39^, ^40^] metal organic vapor deposition MOCVD,[^41^, ^42^, ^43^, ^44^] physical vapor deposition PVD).[^45^, ^46^]

Nevertheless, (Bi_x_Sb_1−x_)_2_Te_3_ ternary solid solutions have demonstrated higher *zT* values than their binary counterparts, mainly due to their larger unit cell size, lower crystal symmetry, and higher site‐occupancy disorder.[^47^, ^48^] For instance, Xie et al. reported a maximum *zT* value of 1.56 at room temperature for a (Bi_0.26_Sb_0.74_)_2_Te_3_ bulk material of low dimensional structure, only to achieve an outstanding enhancement of 50 % in comparison to commercial Bi_2_Te_3_.^[49]^ Similarly, using a combination of ball milling and hot‐pressing techniques Poudel et al. prepared a p‐type nanocrystalline BiSbTe alloy with record‐high *zT*=1.4 at 380 K.^[11]^ More recently, magnetron co‐sputtering methods were employed to fabricate p‐type (Bi_x_Sb_1−x_)_2_Te_3_ thermoelectric thin films of various chemical compositions. Song et al. investigated the influence of the Bi content (x=0‐0.57) on the microstructure and electrical transport properties and found that the grain size of the nanocrystalline (Bi_x_Sb_1−x_)_2_Te_3_ films decreased with higher Bi content. Correspondingly, while the carrier concentration of the films decreased with increasing x value, the Seebeck coefficient increased from 114 to 240 μV K^−1^, leading to a maximum power factor of 31.3 μW K^−2^cm^−1^ for x=0.45.^[50]^ On the other hand, Shang and co‐workers reported the fabrication of a flexible TE generator using p‐type Bi_0.5_Sb_1.5_Te_3_‐based heterostructure films deposited on polyimide substrates. In this work, the random distribution of nanoscale heterojunctions among Bi_0.5_Sb_1.5_Te_3_ and newly introduced phases (Te and Sb_2_Te_3_ nanoinclusions) helped suppress the thermal conductivity (0.8 Wm^−1^ K^−1^) in order to achieve a good power factor of 23.2 μ WK^−2^ cm^−1^.^[51]^

Meanwhile, several groups have also performed low temperature syntheses of (Bi_x_Sb_1−x_)_2_Te_3_ nanoparticles using wet‐chemical based approaches, however very few have reported on the consolidation of the resulting materials for transport measurements.[^48^, ^52^, ^53^, ^54^] Notably, Liu and co‐workers hydrothermally synthesized Bi_0.5_Sb_1.5_Te_3_ nanocrystals by reaction of ECl_3_ (E=Sb, Bi) with elemental Te in the presence of NaBH_4_ as reducing agent; the nanocrystal powders were then consolidated by cold‐pressing and sintering, whereas Dharmaiah and co‐workers applied spark plasma sintering instead.[^55^, ^56^] Unfortunately, the reductive conditions and low thermal stability of traditional precursors [i. e. BiCl_3_, Bi(AOC)_3_, Bi(NMe_2_)_3_] that are typically used in solution‐based methods often lead to Te‐ or Bi‐rich materials due to the incorporation of unwanted metal impurities into the crystal lattice.[^57^, ^58^, ^59^] As a result, off stoichiometric compositions and high number of defects could ultimately poison the Seebeck coefficient and lower the electrical conductivity due to suboptimal changes in the charge carrier concentration.[^9^, ^60^] Even though wet chemical processes offer more control over the size‐ and shape‐selective syntheses of (Bi_,_Sb)_2_Te_3_ nanoparticles, the presence of organic residues stemming from the stabilizing capping agents (i. e. EDTA, OA, OAC, ODT, DDT, PVP) was shown to drastically compromise the electrical conductivity.[^48^, ^56^, ^61^, ^62^] Nonetheless, we successfully demonstrated in our previous works the general applicability of ionic liquids (ILs) not only as an effective solvent medium, but also as a stabilizer and shape‐directing template. Using weakly coordinating ILs, we synthesized Sb_2_Te_3_, Bi_2_Se_3_, Bi_2_Te_3_, and (Bi_x_Sb_1−x_)_2_Te_3_ nanoparticles with extremely low traces of carbon impurities and surface contamination.[^62^, ^63^, ^64^, ^65^] As a result, the materials showed exceptional *zT* values of up to 1.5 due to significant optimization of the electrical conductivity in comparison to commercial powder samples.^[65]^

We herein report on the synthesis of phase‐pure ternary tetradymite‐type materials (Bi_x_Sb_1−x_)_2_Te_3_ (x=0.25, 0.5. 0.75) using a surfactant‐free ionic liquid‐based approach. The alloys series were synthesized by reacting the Bi‐containing ionic liquid [C_4_C_1_Im]_3_[Bi_3_I_12_]^[64]^ with the *single source precursor* (Et_2_Sb)_2_Te and were subsequently subjected to a post‐thermal treatment. This modified route ensures the formation of pure nanoparticles by removing any ionic liquid residues. Aside from the effect of thermal annealing on the chemical composition and nanostructure of the resulting nanoparticles, we herein also report on the effect of the nanoparticle processing on the resulting transport properties. The chemical composition, phase purity, morphology, and particle surface of the obtained nanomaterials were examined by energy‐dispersive X‐ray spectroscopy (EDX), powder X‐ray diffraction (PXRD), scanning electron microscopy (SEM), and transmission electron microscopy (TEM).

## 2 Results and Discussion

### 2.1 Material Synthesis

Low temperature syntheses of tetradymite type materials often require reactive metal organic precursors with well‐defined decomposition pathways to guarantee the formation of highly stoichiometric products. The thermal decomposition of the *single source precursor* (Et_2_Sb)_2_Te[^61^, ^65^] and others[^66^, ^67^] was previously demonstrated as an effective route for obtaining binary Sb_2_Te_3_ nanoparticles with precise chemical composition and defined defect concentration. The low defect and carrier densities of these materials were proven crucial for the optimization of the electronic transport properties, especially the Seebeck coefficient. Similarly, the reactive IL [C_4_mim]_3_[Bi_3_I_12_] was applied in the wet‐chemical synthesis of binary Bi_2_Se_3_ and Bi_2_Te_3_ nanoparticles.^[62]^ The high reactivity yet increased thermal stability of [C_4_mim]_3_[Bi_3_I_12_] compared to many other bismuth precursors eliminated the occurrence of unwanted side reactions such as homolytic bond breakage reactions and formation of Bi double layers. Together, the combined decomposition of these precursors will ensure the production of ternary alloys with the correct stoichiometry and defined concentrations of the metal (Bi/Sb) dopant.

**Scheme 1:**
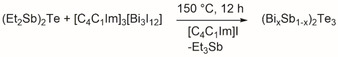
Synthesis of (Bi_x_Sb_1−x_)_2_Te_3_ (x=0.25, 0.5, 0.75) nanoparticles by reaction of (Et_2_Sb)_2_Te with [C_4_mim]_3_[Bi_3_I_12_] in [C_4_C_1_Im]I at 150 °C.

We herein synthesized (Bi_x_Sb_1‐x_)_2_Te_3_ (x=0.25, 0.5, 0.75) nanoparticles from a modified route by reaction of (Et_2_Sb)_2_Te with different amounts of [C_4_mim]_3_[Bi_3_I_12_] in [C_4_C_1_Im]I.^[64]^ Upon thermolysis of (Et_2_Sb)_2_Te at 150 °C, the reaction was stirred for 12 h, after which the resulting metal chalcogenide powders were repeatedly washed with acetonitrile and isolated by centrifugation. All experimental steps including nanoparticle synthesis and purification were strictly performed under inert gas conditions to avoid surface oxidation of the resulting materials (Scheme 1). For high quality materials, the (Bi_x_Sb_1−x_)_2_Te_3_ nanoparticle series were annealed at 250 °C under a vacuum medium of 10^−6^−10^−7^ mbar for 24 h and were then naturally cooled down to room temperature.

### 2.2 Characterization

**Figure 1:**
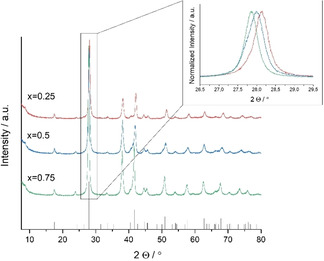
PXRDs of (Bi_x_Sb_1−x_)_2_Te_3_ (x=0.25, 0.5, 0.75) nanoparticles and reference for (Bi_0.5_Sb_0.5_)_2_Te_3_ (PDF 072–1853) shown as black vertical bars.^[69]^

According to PXRD, the as‐prepared (Bi_x_Sb_1‐x_)_2_Te_3_ nanoparticles (x=0.25, 0.5, 0.75) were confirmed as phase‐pure ternary materials (Figure 1). All observed Bragg reflections can be indexed to the rhombohedral (Bi_0.5_Sb_0.5_)_2_Te_3_ (PDF 072–1853, ICSD database). The increasing substitution of the antimony sites by bismuth atoms can be observed from the reflection shifts towards lower reflection angles and from the increase of the lattice parameters. In this case, the gradual exchange of the smaller antimony atoms by larger bismuth atoms would eventually expand the unit cell volume. Most importantly, the steady shift of the highest intensity reflection proves the alloying of a single‐phase material rather than the formation of a separate Bi_x_Te_y_ component.

In addition, the gradual peak broadening of the full width at half‐maximum (fwhm) is consistent with the decreasing size of the crystalline domains in response to the increasing bismuth concentration. Rietveld refinements on the PXRD patterns of (Bi_x_Sb_1−x_)_2_Te_3_ revealed crystallite sizes of 47, 22, and 20 nm for x=0.25, 0.5, and 0.75 using the Scherrer equation (Table 1).^[68]^

**Table 1:** Refined lattice parameters and crystallite sizes of (Bi_x_Sb_1‐x_)_2_Te_3_ nanoparticles. The calculated crystallite sizes should be handled with care due to the anisotropic nature of the samples.

As prepared				
x	a [Å]	b [Å]	V [Å^3^]	Size [nm]
0.25	4.2931	30.5064	486.9	47
0.5	4.3204	30.4601	492.4	22
0.75	4.3491	30.4932	499.3	20

After annealing				
x	a [Å]	b [Å]	V [Å^3^]	Size [nm]
0.25	4.2983	30.5180	488.3	165
0.5	4.3265	30.4705	494.0	95
0.75	4.3477	30.4910	498.8	73

However, the exclusive sharpening of the (110) reflection at 41.61° (2θ), that is observed in the sample containing the highest bismuth content, indicates a preferential growth along the ab‐plane perpendicular to the c‐axis. Shouldering of the (015) and (1010) reflections, which would indicate the presence of elemental Te, are absent in any sample of the alloy series. Other impurity phases including crystalline oxidation products (i. e. Sb_2_O_3_, Bi_2_O_3_, TeO_2_), metal Sb or metal Bi, as well as additional Bi_x_Te_y_ phases were also not observed.

**Figure 2:**
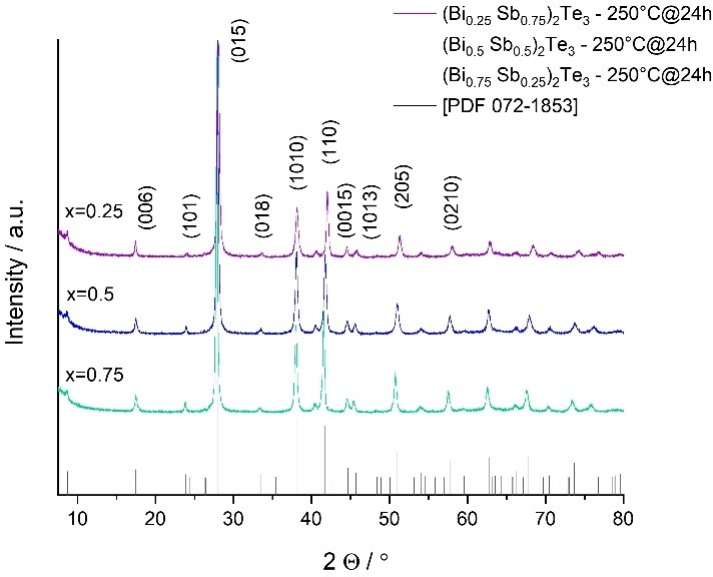
PXRDs of (Bi_x_Sb_1−x_)_2_Te_3_ (x=0.25, 0.5, 0.75) nanoparticles after annealing at 250 °C for 24 h under vacuum and reference for (Bi_0.5_Sb_0.5_)_2_Te_3_ (PDF 072–1853) shown as black vertical bars.^[69]^

Previous studies proved that the processing temperature of post‐thermal treatments may heavily influence the microstructures and transport properties of (Bi_x_Sb_1−x_)_2_Te_3_ based materials by eliminating crystal defects and modifying the chemical composition, grain size, and carrier concentration.[^50^, ^55^, ^60^, ^70^] We herein annealed the (Bi_x_Sb_−x_)_2_Te_3_ nanoparticles in a glass ampoule at a base pressure of 10^−6^−10^−7^ mbar for 24 h. The heat treatment temperature was kept below 300 °C to avoid the excessive evaporation of tellurium, which would otherwise lead to Te‐deficient materials. Upon completion of the heat treatment, a yellow to orange film was deposited on the cold end of the glass wall, which closely resembles the thermochromic behavior of [C_4_mim]_3_[Bi_3_I_12_]. NMR analysis revealed that the thin amorphous film consists of ionic liquid residues including [C_4_mim]_3_[Bi_3_I_12_] and [C_4_C_1_Im]I (Figure S2). As expected, the color intensity and glass coverage of the ionic liquid residue that was extracted from the (Bi_x_Sb_1‐x_)_2_Te_3_ samples increased with higher concentrations of bismuth. The heat treatment serves as a final purification step to remove ionic liquid residues that remained on the particle surface after the washing procedure. The presence of such impurities could especially compromise the electrical mobility of these nanomaterials. The XRD peak assignment of the alloy series after annealing verified the phase purity of these materials as ternary solid solutions of (Bi_x_Sb_1−x_)_2_Te_3_ (Figure 2). The XRD patterns for all annealed samples show no traces of elemental Bi, Sb, or Te and no sign of additional reflexes such as metal oxide phases, which would indicate a possible oxidation of the materials during the annealing process.

The calculated crystallite size of the annealed (Bi_x_Sb_1−x_)_2_Te_3_ nanoparticles is almost 3.5 times larger (Scherrer's equation) compared to the original samples (Table 1). As previously observed, the decreasing crystallite size order of the annealed samples is consistent with the increasing concentration of bismuth (x). When directly comparing the XRD patterns of the alloy series before and after annealing, the narrowing of the fwhm of the annealed samples indicates a higher degree of crystallinity and an increase in the crystallite size.

Quantification of the EDX spectra within standard deviations confirmed the stoichiometric compositions of the ternary alloy series which closely correspond to the theoretical Bi,Sb : Te ratio of 40 : 60 (Table 2). The bismuth to antimony elemental proportions in the sum formula (Bi_x_Sb_1−x_)_2_Te_3_ could be approximated to the desired compositions where x=0.28, 0.51, and 0.65. The EDX results agree with the PXRD measurements, hence confirming the high stoichiometry of the (Bi_x_Sb_1−x_)_2_Te_3_ nanoparticle alloys.

**Table 2:** EDX results from the as prepared and annealed (Bi_x_Sb_1‐x_)_2_Te_3_ nanoparticles.

	As prepared			After annealing		
x	Bi (%)	Sb (%)	Te (%)	Bi (%)	Sb (%)	Te (%)
0.25	11.0±0.1	28.3±0.3	60.7±0.4	11.0±0.1	28.8±0.3	60.2±0.3
0.5	20.3±0.1	19.5±0.2	60.2±0.3	19.9±0.1	19.6±0.4	60.6±0.3
0.75	25.2±0.3	14.3±0.3	60.5±0.3	25.1±0.2	14.4±0.5	60.5±0.3

**Figure 3:**
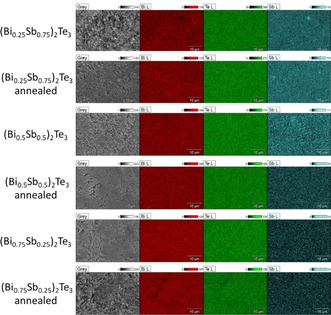
EDX mapping of (Bi_x_Sb_1−x_)_2_Te_3_ (x=0.25, 0.5, 0.75) nanoparticles as‐prepared and after annealing.

EDX elemental mapping analyses revealed a homogeneous distribution of Bi, Sb, and Te within the materials (Figure 3).

**Figure 4:**
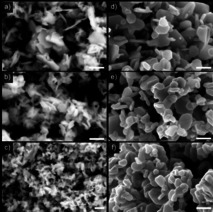
SEM images of (Bi_x_Sb_1−x_)_2_Te_3_ (x=0.25, 0.5, 0.75) nanoparticles as prepared (a‐c) and after annealing (d–f). Scale bar 200 nm.

No significant changes in the material compositions were observed after annealing, which is consistent with the results obtained from PXRD, and no signals originating from ionic liquid contaminations were detected in the EDX measurements. SEM images of the (Bi_x_Sb_1−x_)_2_Te_3_ ternary materials showed the formation of anisotropic nanoparticles with the hexagonal plate‐like morphology (Figure 4). The average edge lengths of the as‐prepared materials are 70, 55, and 53 nm for x=0.25, 0.5, and 0.75, respectively. After the annealing process, the average edge length increased to 97, 87, and 80 nm, respectively, while the average particle thickness increased from 16 to 35 nm.

**Figure 5:**
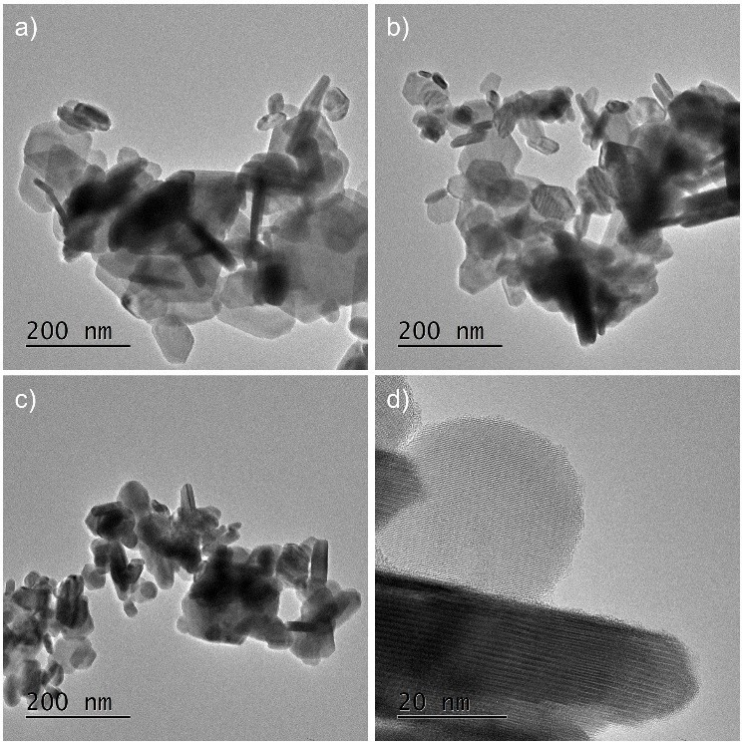
TEM bright field images of (Bi_x_Sb_1‐x_)_2_Te_3_ nanoparticles synthesized by reaction of (Et_2_Sb)_2_Te with [C_4_mim]_3_[Bi_3_I_12_] in [C_4_C_1_Im]I at 150 °C; (a) x=0.25, (b) x=0.5; (c) x=0.75. (d) TEM image of (Bi_0.75_Sb_0.25_)_2_Te_3_ crystallites at higher magnification.

TEM bright field images revealed the hexagonal plate‐like shape of the (Bi_x_Sb_1−x_)_2_Te_3_ nanoparticles ranging from 30 to 200 nm in size and 10 to 20 nm thickness (Figure 5).

**Figure 6:**
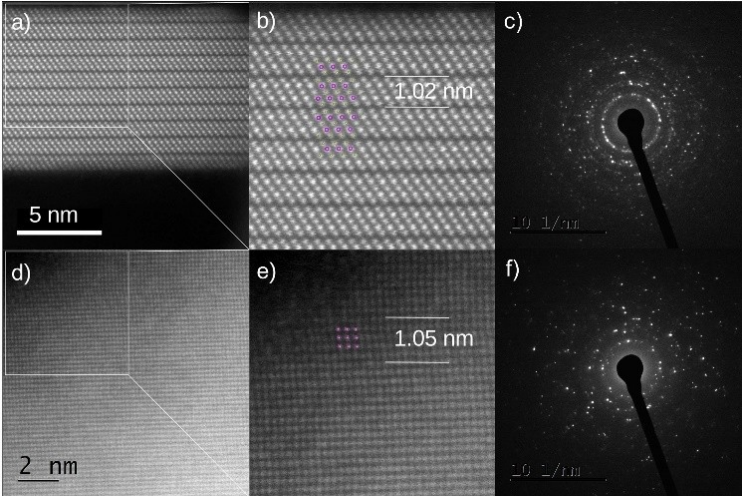
TEM characterization before / after annealing: (a) HAADF STEM images of (Bi_0.75_Sb_0.25_)_2_Te_3_ nanoparticles synthesized by reaction of (Et_2_Sb)_2_Te with [C_4_mim]_3_[Bi_3_I_12_] in [C_4_C_1_Im]I at 150 °C. (b) Overlay of the model crystal structure of (Bi,Sb)_2_Te_3_ (purple: Bi/Sb, yellow: Te). (c) Electron diffraction pattern of (Bi_0.75_Sb_0.25_)_2_Te_3_. (d) HAADF STEM images of (Bi_0.75_Sb_0.25_)_2_Te_3_ nanoparticles after annealing. (e) Overlay of the model crystal structure of (Bi,Sb)_2_Te_3_ (purple: Bi/Sb, yellow: Te). (f) Electron diffraction pattern of (Bi_0.75_Sb_0.25_)_2_Te_3_ after annealing under dynamic vacuum for 24 h C at 250°.

Due to the elimination of shape and size‐directing capping agents, large size distributions of small spherical particles as well as large intergrown plates can be observed in the resulting (Bi_x_Sb_1−x_)_2_Te_3_ samples. High‐resolution TEM measurements (HR‐TEM) confirmed the crystallinity and clean surfaces of the (Bi_x_Sb_1−x_)_2_Te_3_ nanoparticles. In particular, the HR‐TEM dark field image of the (Bi_0.75_Sb_0.25_)_2_Te_3_ ternary phase in [110] orientation displays uniform quintuple layers of 1.02 nm thick, which confirms that there are no additional Bi‐bilayers in the lattice structure before (as‐prepared) and after annealing (Figure 6). Hence, the results prove that the annealing step does not negatively affect the crystallinity of the final materials. Analogous findings were observed for the (Bi_0.25_Sb_0.75_)_2_Te_3_ ternary phase (Figures S4,S5).

Additionally, the SAED pattern of (Bi_0.75_Sb_0.25_)_2_Te_3_ (Figure 5c) once again verifies the crystalline nature of the material. The crystal structure of the resulting (Bi_x_Sb_1−x_)_2_Te_3_ materials is thus proven to adopt a layered and infinite arrangement of quintuple layers (QLs) that are stacked along the c‐axis (Figure S3). In each QL, one could observe five atomic layers with a Te(1)‐Bi‐Te(2)‐Bi‐Te(1) stacking sequence that is terminated by a Te(1) atomic layer on both sides. More specifically, the bonding between the Bi or Sb and Te atoms inside the quintuple QL are best described as strong covalent and ionic interactions, whereas the interactions among the adjacent QLs consist of weak van der Waals forces.^[16]^

### 2.3 Thermoelectric Properties

Thermoelectric properties of alloyed chalcogenides are often optimized by the approach of high energy ball milling followed by rapid compaction like SPS.^[73]^ The alloy composition hereby may even be adjusted purely mechanically during the milling procedure. This approach provides a pragmatic guideline towards high *zT* materials, but makes an assessment of the influences on the thermal conductivity reduction difficult by the multitude of potential sources like alloy composition itself, nanoparticle structure, or other defects that are typically produced by the ball milling. In part, even the amorphization of the nanoparticles by the high energy ball milling may be expected. Motivated by the excellent purity and crystalline integrity of the here‐synthesized nanoparticles, we assess these influences in our compacted nanoparticle pellets. The synthesis and all subsequent processing steps were performed under inert gas conditions, hence avoiding the effect of oxygen impurities. We further optimize the nanoparticle processing steps and discuss the in‐plane and through‐plane thermal conductivity of the obtained nanocrystalline bulk pellets.

### 2.4 Parameters and Reproducibility Study of Hot‐Pressing Procedure

**Figure 7:**
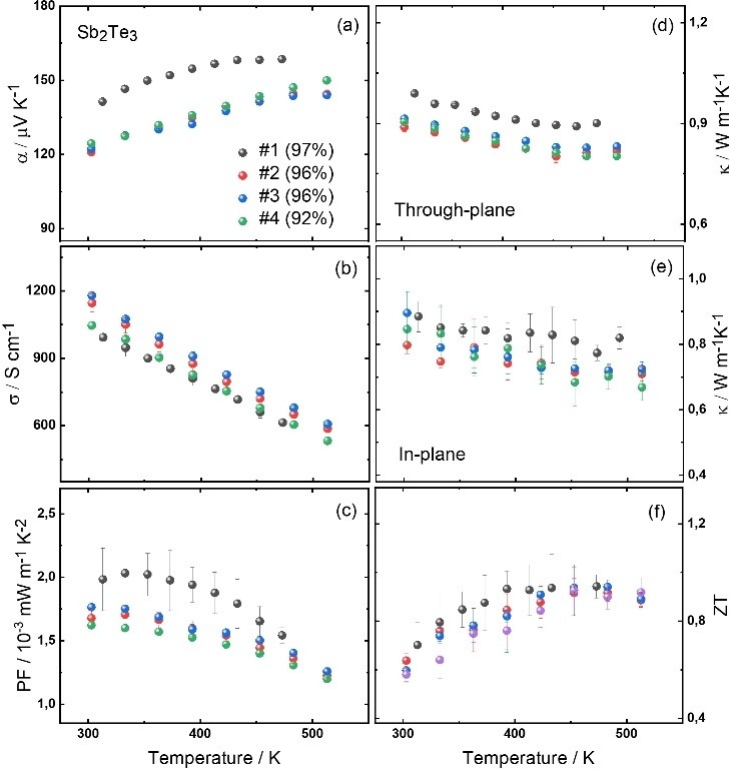
Reproducibility study of hot‐pressing procedure with Sb_2_Te_3_ nanoparticles: a) in‐plane Seebeck coefficient; b) in‐plane electrical conductivity; c) in‐plane power factor; d) through‐plane thermal conductivity; e) in‐plane thermal conductivity; f) in‐plane *zT*.

The limited availability of powder per batch obtained by the chemical synthesis made it necessary to adopt methods and tools appropriately to be able to produce highly dense pellets of small quantities of powder in the order of 70 mg per pellet. Note that it is not common to process this small quantity of nanoparticles, since usually hot pressing or spark plasma sintering is carried out with batches of several grams. 70 mg of the nanoparticles were hot‐pressed at comparatively mild temperature of 300 °C and 100 MPa, being this the optimized parameters of the process with respect to the density of the final product. Therefore, we first performed a reproducibility study of this hot‐pressing procedure to identify the variations from sample to sample and verify stable processing conditions. For reasons of simplicity, we used the binary compound Sb_2_Te_3_ for this study, characterized in‐depth in an earlier work,^[72]^ synthesized by an IL approach that is similar to the one developed for the alloy nanoparticles.^[72]^ A set of hot‐pressed samples of Sb_2_Te_3_ were produced by keeping the processing parameters identical and the complete thermoelectric properties were characterized (Figure 7).

The hot‐pressing procedure resulted in reproducible over‐all properties and almost identical *zT* values (∼1 at 550 K) of all samples within the given error bars. These results are comparable with published data,^[70]^ despite the severely reduced amount of powder and a different kind of processing. Often, variation in the processing of the powder might result in a strong variation of the transport properties such as the thermal conductivity, electrical conductivity and so on. Therefore, we performed this part of the study to emphasize that both synthesis and processing are suitable for revealing the intrinsic properties of the materials rather than existing impurities and defects induced by the processing.

### 2.5 Investigating the Effect of Annealing

**Figure 8:**
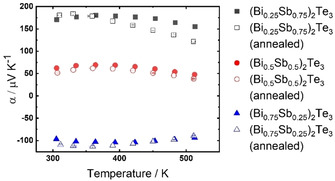
a) Comparison of temperature dependent Seebeck coefficients of the alloy series for (Bi_0.75_Sb_0.25_)_2_Te_3_, (Bi_0.5_Sb_0.5_)_2_Te_3_ and (Bi_0.25_Sb_0.75_)_2_Te_3_ obtained by the optimized hot pressing procedure, with and without annealing step.

The here‐synthesized alloy nanoparticles were in part objected to an annealing procedure. This additional annealing step was intended to verify the cleanliness of the nanoparticles since residual IL could be removed by exposing the nanoparticles to a base pressure of 10^−6^−10^−7^ mbar for 24 h at temperatures below 300 °C. The Seebeck coefficient is the transport coefficient that is most sensitive to any changes in the Fermi level of the semiconductor and therewith the chemical composition of the nanoparticles. Therefore, in Figure 8, we compare the temperature dependent Seebeck coefficients of the nanoparticles of the alloy series, all processed by the optimized hot‐pressing, with and without annealing. It is evident that the annealing procedure does not alter the Seebeck coefficients and therewith the Fermi level of the materials. The trends of the measured Seebeck coefficients represent the expected behavior of this alloy series with respect to the Bi content. This therefore proved as a beneficial additional step in the processing to further purify the surfaces of the nanoparticles, without affecting the chemical composition and electronic band structure that were intentionally designed by the complex synthesis procedure.

### 2.6 Comparison of Nanoparticle Processing by Hot Pressing and Spark Plasma Sintering

The consolidation of the powder may affect the transport properties of the pellet. The sintering affects the microstructure of the macroscopic pellets, but also the reconstruction of the individual interfaces at the atomic level.^[74]^ We here use a bottom‐up approach for the nanoparticle synthesis that emphasizes on structural and crystalline integrity of the nanoparticles. Therefore, the main objective of the processing is compaction rather than reconstruction of the individual grains.

**Figure 9:**
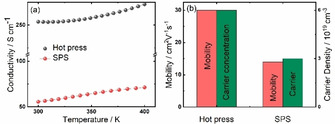
a) Comparison of temperature dependent electrical conductivity of a hot pressed and SPS processed sample of the composition (Bi_0.75_Sb_0.25_)_2_Te_3_. b) Comparison of room temperature charge carrier mobility and charge carrier density of a hot pressed and a SPS processed pellet.

This experiment was designed in a way to directly compare the influence of hot‐pressing and spark plasma sintering. For this, two pellets of the same nanoparticle batch of the composition (Bi_0.75_Sb_0.25_)_2_Te_3_ were produced by the optimized hot‐pressing procedure and a spark plasma sintering procedure, respectively. Because of the different technologies used to densify the nanoparticles, parameters like temperature and pressure had to be adapted. Figure 9a) shows the obtained electrical in‐plane conductivities of the two pellets, together with an analysis of carrier mobility and carrier density (Figure 9b). Hereby, the electrical conductivity provides a good measure for this comparison since it is sensitive towards the quality of crystalline interfaces and grain boundaries. Both samples differ in their density: The hot‐pressing procedure resulted in a density of 6 g cm^−3^ (81 %), while the SPS processed sample showed only a density of 5.1 g cm^−3^ (70 %) after the typical signatures of sample shrinking detected within the SPS machine by tracking the movement of the tools. Note that higher temperatures or longer hold times realized after the observation of sample shrinking typically only result in partial melting.^[71]^ As a result, it was found that the hot‐pressing procedure resulted in an electrical conductivity of around 250 S cm^−1^ at RT that was higher than the electrical conductivity of 50 S cm^−1^ of the SPS sample. This was caused by both, an improved charge carrier density as well as an improved charge carrier mobility of this n‐type alloy sample. We conclude that the hot‐pressing procedure generates the conditions for an efficient thermal activation of carriers in the pellet. Further, defects, especially pores, that reduce the carrier mobility are less favored by the hot‐pressing than by the SPS processing. Hence, hot‐pressing is better suitable to allow for a dense arrangement of the here‐used nanoparticles in the compacted pellet, also resulting in improved electrical properties.

### 2.7 Thermal Conductivity of the Alloy Series

The parts of the study presented above were all intended to develop a reliable processing for small quantities of nanoparticles. By the processing in a glove box cluster, we avoid the incorporation of unwished oxygen impurities. The hot‐pressing provides a high density and high reproducibility of the obtained pellets (Figure 7). The additional annealing of the nanoparticles improves the purity with respect to potential incorporation of residues of the IL, without affecting the chemical composition (Figure 8). Therefore, the combination of the sophisticated nanoparticle synthesis combined with this processing provides a means to understand the influence of the nanoparticle alloying on the thermal conductivity in‐plane and through‐plane of the pellets. Influences that are typically found in alloyed nanoparticles like the incorporation of defects from mechanical alloying by ball milling or the incorporation of impurities by the chemical synthesis are not expected to play a major role here.

**Figure 10:**
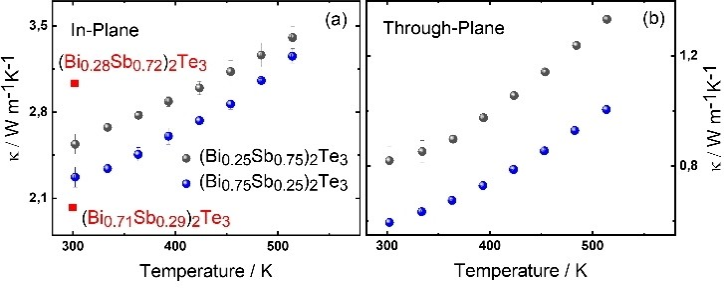
a) In‐plane thermal conductivity and b) through‐plane thermal conductivity for (Bi_0.75_Sb_0.25_)_2_Te_3_ and (Bi_0.25_Sb_0.75_)_2_Te_3_ obtained by the optimized hot‐pressing procedure, with additional annealing step. Single crystalline reference data from ref. [75] added.

Figure 10 shows the in‐plane and through‐plane temperature dependent thermal conductivity of annealed nanoparticles of the composition (Bi_0.75_Sb_0.25_)_2_Te_3_ and (Bi_0.25_Sb_0.75_)_2_Te_3_. The change in the thermal conductivity with Bi content is consistent with what is expected from previous literature.^[75]^ With less Bi content, the thermal conductivity increases. Clearly, the thermal conductivity of the pellets exhibits an anisotropy indicating that the nanoparticles align within the pellets with a preferred orientation of the crystallographic ab‐plane in‐plane of the pellets. This was found before for similarly synthesized and compacted Bi_2_Te_3_ and Bi_2_Se_3_ nanoparticles.^[62]^

Further we find that the thermal conductivity is higher than expected for nanocrystalline bulk of this alloy compositions. Single crystalline reference data of similar compositions is added to Figure 10. Hence, the thermal conductivity of our samples is in‐between the single crystalline reference and nanocrystalline bulk of other processes. For instance, for the alloy composition (Bi_0.25_Sb_0.75_)_2_Te_3_ a ball milling and hot‐pressing approach resulted in isotropic samples, which showed thermal conductivities of around 1 W m^−1^ K^−1^ at room temperature. While this value corresponds to the here‐presented through‐plane direction, it is significantly reduced compared to the thermal conductivity in in‐plane direction,^[73]^ and would not represent the isotropic average over all crystalline directions. For (Bi_0.7_Sb_0.3_)_2_Te_3_, the in‐plane thermal conductivity was found to be around 1.3 W m^−1^ K^−1^ at room temperature for a sample that was also exposed to a combination of ball milling and SPS.^[70]^ A recent report on a micro‐wave assisted solution‐synthesis of (Bi_x_Sb_1−x_)_2_Te_3_ nanoparticles followed by SPS processing^[37]^ yielded pellets, which featured a low thermal conductivity of below 1 W m^−1^ K^−1^ at room temperature for a composition of (Bi_0.25_Sb_0.75_)_2_Te_3_. This comparison shows that the thermal transport properties of the obtained product are not mainly defined by the chemical composition, but rather by the sum of the synthetic details such as the used additives, as well as the processing details including the compaction method. In an earlier study, we already showed that high purity Bi_2_Te_3_ and Bi_2_Se_3_ compacted nanoparticles exhibited an exceptionally high thermal conductivity close to that of single crystals or even ab‐initio calculations for bulk.^[62]^ We now showed for the alloyed nanoparticles a consistent behavior. Obviously, the often‐reported low thermal conductivity of samples prepared by ball milling followed by rapid compaction originates from the morphological peculiarities like point defects or dislocations rather than the chemical composition or the nanostructure itself.

While the here‐synthesized nanoparticles provide a close‐to‐perfect model system to study the influence of chemical composition and nanostructure on the transport properties, they obviously cannot compete with the best thermoelectric materials of the nominally identical composition due to their high thermal conductivity which results from the extraordinary high purity and crystalline integrity of the nanoparticles. Consequently, *zT* values of these alloyed nanoparticles range from *zT*=0.06 to *zT*=0.15 at 400 K for the composition of (Bi_0.25_Sb_0.75_)_2_Te_3_ in in‐plane direction, depending on the details of the applied processing.

## 3 Conclusions

Highly stoichiometric, phase‐pure ternary solid solutions of the type (Bi_x_Sb_1−x_)_2_Te_3_ were synthesized by thermolysis of the *single source precursor* (Et_2_Sb)_2_Te with various amounts of the Bi source [C_4_mim]_3_[Bi_3_I_12_] in a surfactant‐free ionic liquid‐based approach in [C_4_mim]_3_I. The phase purity as well as the perfect stoichiometric composition and homogenous element distribution within the resulting nanoparticles was proven by XRD, SEM, EDX and TEM. After compaction of the nanoparticles, the thermoelectric transport properties, Seebeck coefficient, electrical conductivity and thermal conductivity were determined. Our studies clearly proved the crucial role of nanoparticle processing on the resulting transport properties.

## Experimental Section

### Experimental Details

Nanoparticle synthesis, thermolysis experiments, fabrication of pellets, and electrical contact preparations were performed under inert conditions (Ar atmosphere) in a glovebox or using standard Schlenk techniques to avoid any oxidation reactions. The powders and pellets were carried using sealed and Ar filled vessels until the materials were finally transferred to the measurement devices. Solvents were dried over CaH_2,_ stored over molecular sieves, and degassed prior to use. 1‐N‐Methylimidazole (99 %, Sigma Aldrich), 1‐halobutanes (99 %, Acros) and CH_3_CN (99.9+%, Acros) were commercially available, while [C_4_C_1_Im]I, [C_4_C_1_Im]_3_[Bi_3_I_12_], and (Et_2_Sb)_2_Te were prepared by literature methods.[^64^, ^76^]

### Synthesis of Ternary (Bi_x_Sb_1−x_)_2_Te_3_ Nanoparticles

The corresponding amount (see Table 3) of [C_4_C_1_Im]_3_[Bi_3_I_12_] was dissolved in 5 mL of [C_4_C_1_Im]I, the red ionic liquid solution was stirred under an Ar atmosphere at 150 °C. 1028 μl (4.1 mmol) of (Et_2_Sb)_2_Te were added dropwise yielding a black suspension which was stirred at 150 °C for 12 h. The resulting black precipitate was centrifuged and repeatedly washed with 20 mL of dry acetonitrile (6x). The solid product was dried in vacuum at ambient temperature. An aliquot was transferred to a closed ampoule to be annealed at 250 °C for 24 h under dynamic vacuum (10^−7^ mbar).

**Table 3:** Corresponding amounts of [C_4_C_1_Im]_3_[Bi_3_I_12_] reacted with (Et_2_Sb)_2_Te to synthesize (Bi_x_Sb_1−x_)_2_Te_3_ alloy series.

x	m [mg]	n [mmol]
0.25	877	0.34
0.5	1754	0.68
0.75	2631	1.02

### Thermal Analyses

TGA/DTA studies were performed using a Mettler‐Toledo DSC Star1 system that is operated under inert gas conditions.

### NMR Spectroscopy

^1^H (400 MHz) and ^13^C{1H} (75.5 MHz) NMR spectra (δ in ppm) were recorded using a Bruker AVNEO 400 MHz spectrometer and were referenced to internal DMSO‐d_6_ (^1^H: δ=2.50; ^13^C: δ=39.52).

### Powder X‐Ray Analysis

PXRD patterns were collected with a Bruker D8 Advance diffractometer with Cu Kα radiation (λ: 1.5418 Å, 40 kV, 40 mA) using a Si single crystal as sample holder to minimize scattering. The powder samples were re‐dispersed in EtOH on the Si surface and investigated in a 2θ range from 10 to 90° with a step size of 0.01° 2θ (counting time 0.6 s). Rietveld refinement was done with the program package TOPAS 4.2 (Bruker) to determine lattice parameters and average crystallite sizes by using the Scherrer equation.^[68]^ The background was modelled using Chebyshev polynomials. The structure model of Bi_2_Te_3_ (PDF 15–863) from the ICSD database was used. For each Rietveld refinement, the instrumental correction determined using a standard powder sample LaB_6_ from NIST (National Institute of Standards and Technology) as reference material (SRM 660b; a(LaB_6_)=4.15689 Å) was considered.

### Electron Microscopy

The particle morphology and elemental composition of the powdered samples were analyzed by scanning electron microscopy (SEM) using Apreo S Lovac microscopes equipped with a Bruker Quantax 400 units (EDX). TEM studies were carried out on a Jeol JEM 2200 fs microscope equipped with probe‐side Cs‐corrector operated at 200 kV acceleration voltage.

### Thermoelectric Properties

Pellets were prepared using a manual hydraulic press (Specac Atlas) and a custom‐made heating mantle equipped with a PID temperature control. A pressure of 100 MPa was applied for 90 minutes while the temperature of the stainless‐steel die was kept at 300 °C. One pellet was sintered by spark plasma sintering procedure using SPS 210‐Gx (AGUS). For sintering, a pressure of 35 MPa was applied for 1 minute at temperature of 240 °C. The density of the pellets was determined geometrically. Thermoelectric properties were measured from room temperature up to 240 °C. The thermal diffusivity was measured along the pressing direction and perpendicular to the pressing direction (Linseis LFA). Thermal conductivity was calculated from the diffusivity data with the relation κ=*D* ρ *c*_p_ where *D* is the thermal diffusivity, ρ the density, and *c*_p_ the heat capacity. Bulk literature values were used for *c*_p_.^[70]^ The Seebeck coefficient and the electrical conductivity were measured perpendicular to the pressing direction (Linseis LSR‐3). The majority carrier concentration and carrier mobility were determined perpendicular to the pressing direction at room temperature with Hall measurements in the van‐der‐Pauw geometry (Quantum Design Versalab).

**Supporting Information**: PXRDs including the Rietveld refinement of (Bi_x_Sb_1‐x_)_2_Te_3_ (x=0.25, 0.5, 0.75) nanoparticles, crystal structure of (Bi_x_Sb_1‐x_)_2_Te_3_ materials, and ^1^H‐NMR spectrum of ionic liquid contamination from nanoparticle annealing.

## Conflict of interest

The authors declare no conflict of interest.

## Supporting information

As a service to our authors and readers, this journal provides supporting information supplied by the authors. Such materials are peer reviewed and may be re‐organized for online delivery, but are not copy‐edited or typeset. Technical support issues arising from supporting information (other than missing files) should be addressed to the authors.

Supplementary
