# Supplementary material for: Influence of Nanoparticle Processing on the Thermoelectric Properties of (BixSb1−X)2Te3 Ternary Alloys
Source: ChemistryOpen. 2021 Jan 25;10(2):189–98. doi: 10.1002/open.202000257 (PMC7874259; doi:10.1002/open.202000257)
Supplement: Supplementary file 1 — Supplementary [file OPEN-10-189-s001.pdf]

ChemistryOpen

Supporting Information

Influence of Nanoparticle Processing on the Thermoelectric Properties of $(Bi_xSb_{1-X})_2Te_3$ Ternary Alloys

Sarah Salloum, Georg Bendt, Markus Heidelmann, Kateryna Loza, Samaneh Bayesteh, M. Sepideh Izadi, Patrick Kawulok, Ran He, Heike Schlörb, Nicolas Perez, Heiko Reith, Kornelius Nielsch, Gabi Schierning,* and Stephan Schulz*

Supporting Information

Content

Figure S1. PXRD including Rietveld refinement of $(Bi_xSb_{1-x})_2Te_3$ (x = 0.25, 0.5, 0.75) **S2**

Figure S2. ^{1}H-NMR spectrum of ionic liquid contamination. **S3**

Figure S3. Schematic crystal structure of $(Bi/Sb)_2Te_3$ materials. **S3**

Figure S4. (a) HAADF STEM images of $(Bi_{0.25}Sb_{0.75})_2Te_3$ nanoparticles synthesized by reaction of $(Et_2Sb)_2Te$ with $[C_4mim]_3[Bi_3I_{12}]$ in $[C_4C_1Im]I$ at 150°C. (b) Overlay of the model crystal structure of $(Bi,Sb)_2Te_3$ (purple: Bi/Sb, yellow: Te). **S4**

Figure S5. (a) HAADF STEM images of $(Bi_{0.25}Sb_{0.75})_2Te_3$ nanoparticles after annealing under dynamic vacuum for 24 h C at 250°. (b) Overlay of the model crystal structure of $(Bi,Sb)_2Te_3$ (purple: Bi/Sb, yellow: Te). **S4**

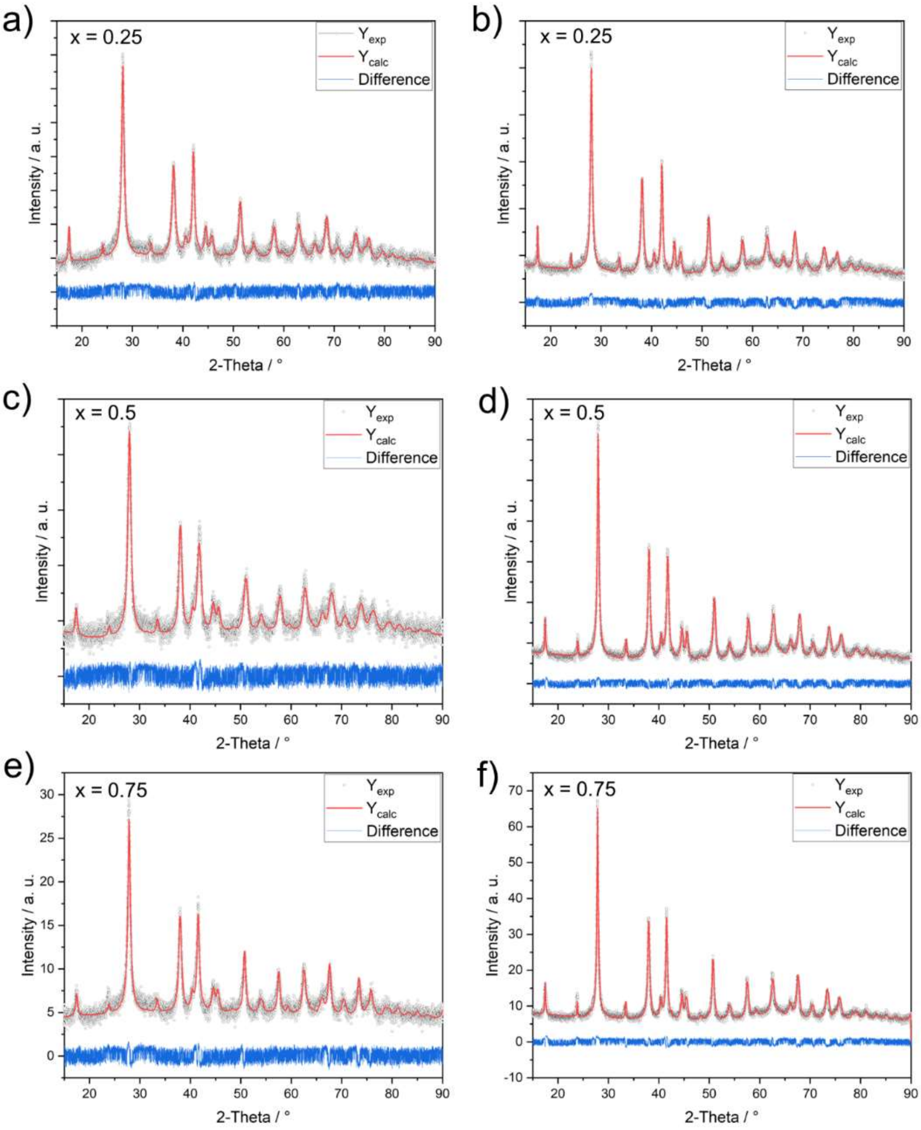

Figure S1. PXRDs including Rietveld refinement of $(Bi_xSb_{1-x})_2Te_3$ powders (x = 0.25, 0.5, 0.75) synthesized by reaction of $(Et_2Sb)_2Te$ with $[C_4mim]_3[Bi_3I_{12}]$ in $[C_4C_1Im]I$ at 150 °C and measured as prepared (a-c) and after annealing (d-f).

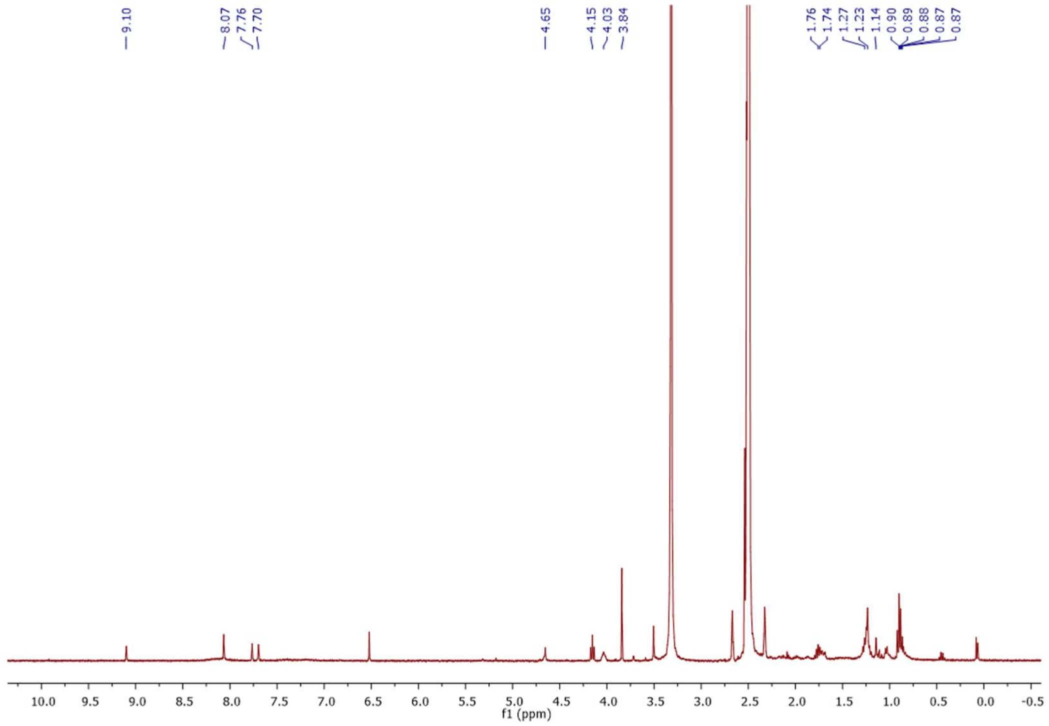

Figure S2. ^{1}H-NMR spectrum of amorphous IL impurities (DMSO-d_6) which were removed from the samples during the annealing process.

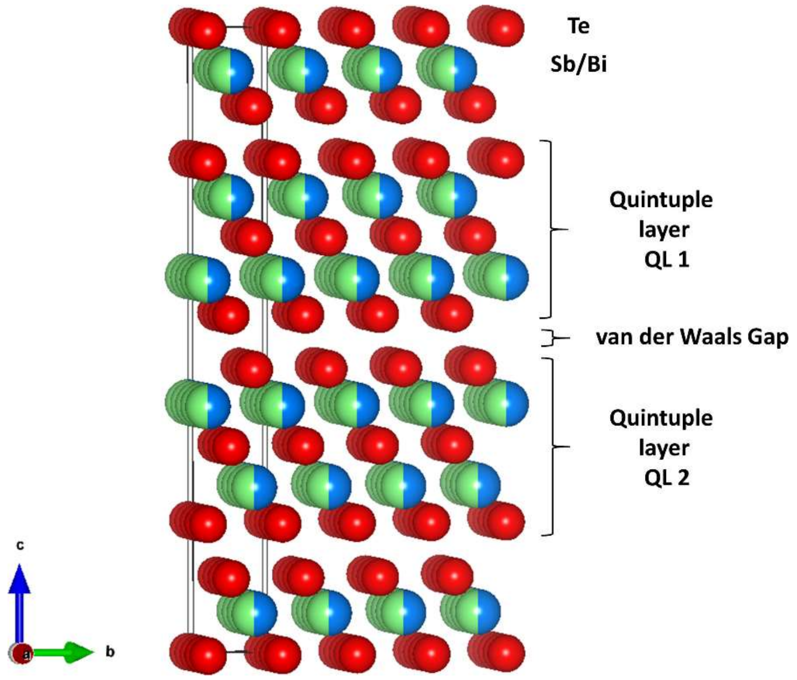

Figure S3. Schematic illustration of the rhombohedral lattice of $(Bi/Sb)_2Te_3$ and stacking of the alternating Te_1–Bi/Sb–Te_2–Bi–Te_1 layers, also known as quintuple layers. [ICSD 20070] M.M. Stasova, N. Abrikosov, *Kh. Izvestiya Akademii Nauk SSSR, Neorganicheskie Materialy,* **1970**, *6*, 1090-1094.

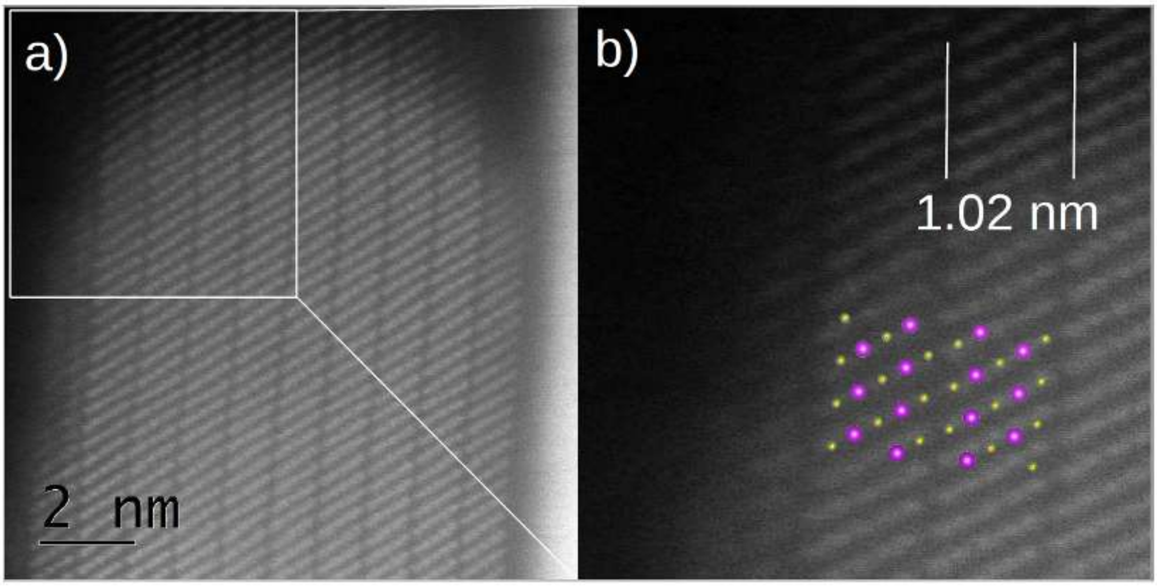

Figure S4. (a) HAADF STEM images of $(Bi_{0.25}Sb_{0.75})_2Te_3$ nanoparticles synthesized by reaction of $(Et_2Sb)_2Te$ with $[C_4mim]_3[Bi_3I_{12}]$ in $[C_4C_1Im]I$ at 150 °C. (b) Overlay of the model crystal structure of $(Bi,Sb)_2Te_3$ (purple: Bi/Sb, yellow: Te).

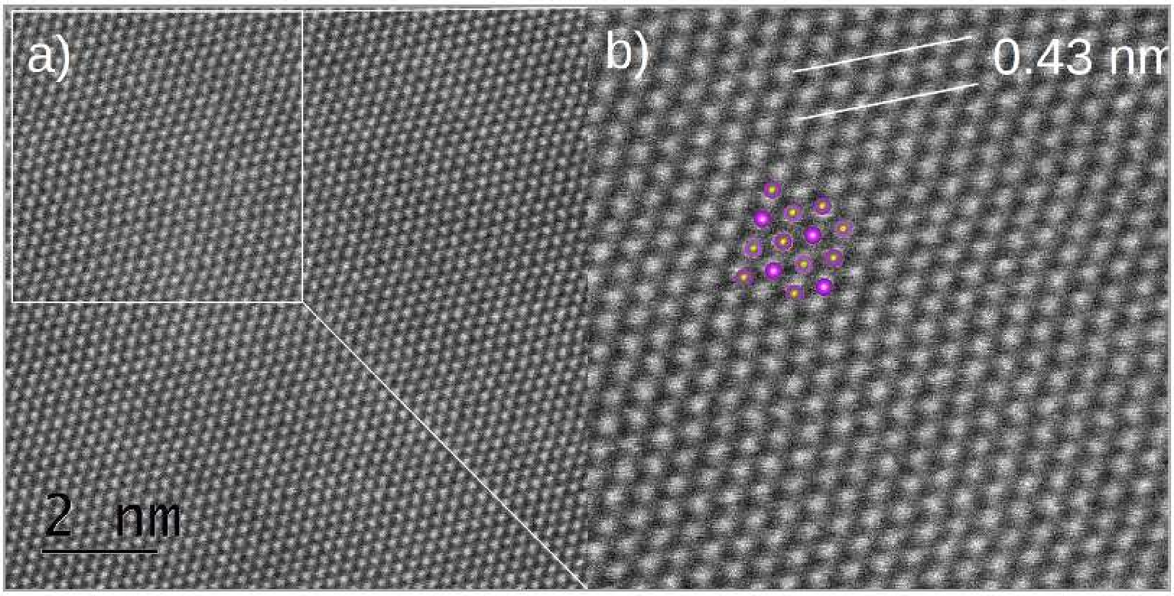

Figure S5. (a) HAADF STEM images of $(Bi_{0.25}Sb_{0.75})_2Te_3$ nanoparticles after annealing under dynamic vacuum for 24 h C at 250 °C. (b) Overlay of the model crystal structure of $(Bi,Sb)_2Te_3$ (purple: Bi/Sb, yellow: Te).
